# Role of Membrane Lipids in the Regulation of Erythrocytic Oxygen-Transport Function in Cardiovascular Diseases

**DOI:** 10.1155/2016/3429604

**Published:** 2016-10-30

**Authors:** Victor V. Revin, Natalia V. Gromova, Elvira S. Revina, Maria I. Martynova, Angelina I. Seikina, Nadezhda V. Revina, Oksana G. Imarova, Ilia N. Solomadin, Alexander Yu. Tychkov, Nikolai Zhelev

**Affiliations:** ^1^Federal State-Financed Academic Institution of Higher Education “National Research Ogarev Mordovia State University”, Saransk 430005, Russia; ^2^GBUZ RM “National Hospital for War Veterans”, Saransk 430005, Russia; ^3^CMCBR, Abertay University, Dundee DD1 1HG, UK

## Abstract

The composition and condition of membrane lipids, the morphology of erythrocytes, and hemoglobin distribution were explored with the help of laser interference microscopy (LIM) and Raman spectroscopy. It is shown that patients with cardiovascular diseases (CVD) have significant changes in the composition of their phospholipids and the fatty acids of membrane lipids. Furthermore, the microviscosity of the membranes and morphology of the erythrocytes are altered causing disordered oxygen transport by hemoglobin. Basic therapy carried out with the use of antiaggregants, statins, antianginals, beta-blockers, and calcium antagonists does not help to recover the morphofunctional properties of erythrocytes. Based on the results the authors assume that, for the relief of the ischemic crisis and further therapeutic treatment, it is necessary to include, in addition to cardiovascular disease medicines, medication that increases the ability of erythrocytes' hemoglobin to transport oxygen to the tissues. We assume that the use of LIM and Raman spectroscopy is advisable for early diagnosis of changes in the structure and functional state of erythrocytes when cardiovascular diseases develop.

## 1. Introduction

Nowadays diseases of the cardiovascular system occupy a leading place among the major causes of disabilities and mortality of the active labour force not only in Russia but throughout the world [1, 2]. Notwithstanding the introduction of new highly effective methods of diagnosis and treatment into clinical practice, mortality from ischemic heart disease (IHD) remains high. Among the IHD risk factors, one of the first causes rests with arterial hypertension, which for its prevalence is called a “disease of civilization.” The most important and frequent manifestation of IHD is angina pectoris.

The pathophysiology of most cardiovascular diseases attributes one of the key places to hypoxia, which causes disorders of the gas-transport function of blood and, in many cases, reduces the efficiency of oxygen transport carried out by erythrocytes. The primary reason is attributed to the disorders in structure and function of endothelium of blood vessels, while the role of erythrocytes and their oxygen-transport ability in the development of vascular diseases remains insufficiently studied [2, 3].

Oxygen-transport function of erythrocytes in peripheral blood depends on many factors, where of great importance is the change in conformation of hemoglobin (Hb) and its affinity to oxygen (O_2_). The use of Raman spectroscopy (RS) made it possible to identify changes in the conformation of hemoporphyrin in patients with severe arterial hypertension and under the therapeutic interval hypoxia in patients with stable effort [4] and in patients with circulatory failure [5–7].

It is important to highlight that oxygen-binding and oxygen-transport properties of hemoglobin depend on the morphological and functional condition of erythrocytic membranes [8, 9].

Current researches pay little attention to one of the most important barriers of oxygen to hemoglobin, that is, the lipid bilayer of erythrocytic membranes, which depends on individual phospholipids, the degree of oxidation, and the ratio of saturated and unsaturated fatty acids. Also insufficiently studied is the mechanism of oxygen transport through the lipid bilayer. And it should be emphasised that a significant part of phospholipids and their metabolites is involved in the regulation of various functions through receptor systems [10–12].

A special role is assigned to oxidative stress being developed in case of lipidic pathologies and the substrates of such development are unsaturated fatty acids. Modification of the composition and structural arrangement of phospholipids in cell membranes may cause disruption of ion transport [13, 14].

Based on this far incomplete list of lipids roles in cell's condition it can be assumed that disorders in lipid composition and condition of erythrocyte membranes play a key role in the development of many cardiovascular diseases [15, 16].

Structural changes of erythrocytic membranes, including the change in mobility of membrane proteins, causes the increased susceptibility to proteolysis [17, 18].

When cardiovascular disease develops it is the cytoarchitectonics of formed elements that underlies the changes in the structural-functional condition of erythrocytic membranes in healthy people as well as among the diseased. Assessment of surface changes in cytoarchitectonics may serve as an indicator of the efficient therapy [7].

It is of great current interest and importance from a practical point of view to study the erythrocyte cytoarchitectonics and its rheological properties as an integral indicator of the development of angina pectoris.

It is yet to be firmly concluded whether there is a relationship between disorders in lipid composition of membranes, changes of morphometric parameters of erythrocytes, and conformational changes of hemoglobin hemoporphyrin in norm and in pathological conditions associated with the development of hypoxia in the body which remains not thoroughly investigated. Considering the above, the aim of our research was to examine changes in lipid composition of membranes, cytoarchitectonics of erythrocytes, conformation of hemoglobin hemoporphyrin and its oxygen-transport function in respect to the clinical course of angina pectoris, and the presence of primary arterial hypertension in patients' anamnesis.

## 2. Materials and Methods

The research was carried out following the approval of the Local ethics Committee of Mordovia State University on the basis at the Mordovian Republican Hospital of War Veterans in accordance with the principles of Good Clinical Practice. Having obtained informed consent for participation in the research, we examined 39 patients at the cardiology department, all males aged from 46 to 63 years (average age of 54.5 ± 3.8 years). Further inclusionary criteria required patients to be nonsmokers, with no history of hereditary anomalies and a body mass index ranging from 23 to 29. Patients were divided into 2 groups depending on disease:

 Group 1—stable angina, *n* = 21;
 Group 2—stable angina on the top of primary arterial hypertension (stage 3, risk 3), *n* = 18.

The research did not include patients with a history of liver, kidney, lung disease, diabetes, systemic diseases, or malignancy.

The treatment was administered using a unified scheme and antianginal drugs, ACE inhibitors, angiotensin II antagonists, beta-blockers, calcium antagonists, electrolytes, antiaggregants, statins, and diuretics. The examination was carried out before and on the tenth day of treatment.

The control group consisted of 19 apparently healthy male donors of the republican station of blood transfusion of a similar age (the average age being 50.5 ± 1.9 years), without evidence of CVD during preventative examinations.

Research results were obtained from patients' blood samples, aseptically taken from the cubital vein in the volume of 5 mL in a fasting state.

Erythrocytes were obtained by centrifugation of whole blood at 1500 ×g for 10 min; then a small portion of sediment was resuspended in the washing fluid; the latter was used for microscopy, while the other part was used to obtain membranes.

To isolate membranes from human erythrocytes sediments of erythrocytes were hemolysed in a cooled to 0°C solution of 5 mM NaH_2_PO_4_; 0.5 mM PMSF (phenylmetylsulfonylfluoride) pH 8.0 (lysis solution). The ratio of erythrocyte sediment to lysis solution was 1 : 20 (V : V).

The mixture was left for 10 min at 4°C and then centrifuged at 20000 ×g for 40 minutes (temperature 0°C). The supernatant was removed; the sediment was resuspended in lysis solution and centrifuged at 20000 ×g for 40 minutes (temperature 0°C). The procedure was repeated three times.

Extraction of membrane lipids was performed by using Bligh and Dyer's method [19]. To separate phospholipids we used one-dimensional chromatography in the system of solvents: chloroform/methanol/water/ammonia (60/34/4/2) [20, 21] and chloroform/methanol/glacial acetic acid/water (60/50/1/4) [22].

The resulting phospholipids were eluted with a mixture of chloroform : methanol (2 : 1). Methylation of fatty acids was performed by using Morrison and Smith's method [23]. Separation of methyl esters of fatty acids was performed on a gas chromatograph GC-2010 Plus (Shimadzu, Japan). The software package GCsolution, Shimadzu, was used. The coefficient of saturation was calculated as the ratio between the sum of saturated and unsaturated fatty acids.

The structure of erythrocytes and hemoglobin content was found by method of laser interference microscopy (LIM) in vitro [24–30] with the use of device MИИ-4M (Russia). The sediment (deposit) of erythrocytes after centrifugation was diluted 20 times with washing medium (10 mm of KH_2_PO_4_, 3.5 mm of KCl, 1.5 mm of MgCl_2_, 145 mm of NaCl, and 6 mm of glucose, pH 7.4). 1 mcL of the erythrocytes' suspension was placed on a mirror glass; a smear was prepared and covered with cover glass. The measurements were carried out at room temperature (18–22°C). For LIM examination we received image of 10 sites with a monolayer arrangement of cells in the interference channel and the reflected light in each sample. The resulting images of red blood cells were processed using FIJI [31].

The condition of human erythrocytes was assessed by registering the mean value of optical path difference (OPD) and the phase image area of erythrocyte, the value of which was calculated using FIJI software [31]. To get a reliable result, indicators were calculated, feeding upon at least 100 cells from each sample.

Additionally we had to evaluate the phase volume of erythrocyte by the formula below:

(1) $$\begin{array}{c} V_{cell}=\frac{\Phi_{mean}S}{n_{cell}-n_{m}}, \end{array}$$

where *S* is the phase image area of erythrocyte, Φ_mean_ is mean value of the measured parameter, optical path difference (OPD), proportional to the thickness of erythrocyte, *n*_cell_ is erythrocyte refraction index equal to 1,405, and *n*_*m*_ is refraction index of normal saline that was equal to 1,333.

Conformation and properties of hemoglobin were defined by Raman spectroscopy on the device In Via Renishaw (UK) with short-focus extreme aperture lens monochromator (focus distance not more than 250 mm) [4, 9, 32, 33]. For excitation of Raman spectra we used the laser with wavelength 532 nm, maximum power 100 mW, and objective 100x and CCD detector for recording data (1024 × 256 pixels with Peltier cooling to –70°C), 1800 lines/mm. The digitised spectra were processed with WIRE 3.3. Reference line correction and smoothing of the spectra were carried out.

The erythrocytes suspension smear was studied on the object glass. Hemoglobin spectra bands were traced out at excitation by laser 532 nm and correlation of bands with variations of porphyrin links was carried out. For each sample the measurements were taken three times and the resulting values were averaged. The position and intensity of Raman scattering (RSc) bands of hemoglobin spectrum depend on variations of links in a porphyrin ring that allows you to estimate the conformation of hematoporphyrin (HP), which is directly related to hemoglobin's oxygen-binding properties [32].

To analyse the conformation of hemoglobin's hematoporphyrin (HP) we took into account specific bands of RS spectrum, which allowed us to explore the conformation of HP in deoxyhemoglobin (d–Hb) and the ability of d-Hb to bind ligands, as well as the HP conformation in oxyhemoglobin (o–Hb) and the ability of o-Hb to reduce the oxygen.

In this paper to analyse the conformation and Hb's О_2_–binding properties we drew on the following RS band spectra of blood (maximum positions indicated): 1355, 1375, 1550, and 1580 cm^−1^.

The use of RS-peaks correlations and not their absolute values is explained by the fact that the absolute value of spectrum intensity depends on the amount of hemoglobin and thus amount of erythrocytes in the sample and in the laser's focus. Internal normalisation of peaks (on the intensity of other bands) ensures that the analysed parameters in different samples do not bear on the amount of hemoglobin and are determined only by the conformation of hemoglobin and the relative content of its various forms.

The character of spectra in Raman scattering (RSc) of hemoprotein hemoglobin [30, 33] allows us to determine the degree of oxidation of the iron atom included, its spin state, and the presence of ligands and reflects changes in the structure of globin, leading to deformation of hemoprotein and affecting oxygen-binding properties of hemoglobin [6].

The intensity of the spectrum bands 1355 and 1375 cm^−1^ is related to symmetrical oscillations of pyrrole rings in deoxyhemoglobin molecules and hemoglobin bound with ligands, respectively [5]. As the amount of O_2_ in blood is 3-4 orders higher than the concentrations of other ligands (e.g., NO or CO), the intensity of the band 1375 cm^−1^ is mainly determined by the content of oxyhemoglobin. Therefore, the intensity ratio *I*_1375_/(*I*_1355_ + *I*_1375_) is proportionate to the relative amount of oxyhemoglobin in blood. Intensities of bands 1550 cm^−1^ and 1580 cm^−1^ characterise the spin state of iron in its deoxy- and oxyform, respectively, and thus are a marker, which assesses the structural characteristics of iron in a prosthetic group.

This makes possible, using the relationship between the intensities of bands *I*_1355_/*I*_1550_ and *I*_1375_/*I*_1580_, evaluating the ability of molecules of hemoglobin in erythrocytes to bind and to release oxygen molecules, respectively, with account to the internal state of hemoglobin molecules. Dividing one ratio by another (*I*_1355_/*I*_1550_)/(*I*_1375_/*I*_1580_) one can obtain a parameter reflecting the affinity of the hemoglobin molecule to oxygen in native erythrocytes [9, 30, 32–35].

Statistical processing of experimental results was carried out in several stages. The first phase evaluated the normality of the distribution of values for each of the samples; we used Geary's criterion [36]. The second stage has assessed homogeneity of variance. Then ANOVA and ANOVA for repeated measures were conducted. In the case of statistically significant differences between the mean values, we used post hoc analysis method of comparison of means by Tukey [37].

The research results are presented as arithmetic means ± standard deviation (means ± SD).

## 3. Results

The findings showed that erythrocytic membranes of healthy people have 5 fractions of phospholipids (PL): sphingomyelin (SM), phosphatidylcholine (PC), phosphatidylserine (PS), phosphatidylinositol (PI), phosphatidylethanolamine (PEA), and diacylglycerol (DAG). The composition of phospholipids' fatty acids (FA) of erythrocytic membranes included myristic (14:0), palmitic (16:0), palmitoleic (C16:1), stearic (C18:0), oleic (C18:1), linoleic (C18:2), linolenic (C18:3), arachidic (C20:0), gondoinic (C20:1), eicosadienoic (C20:2), and behenic (C22:0) acids.

**Figure 1:**
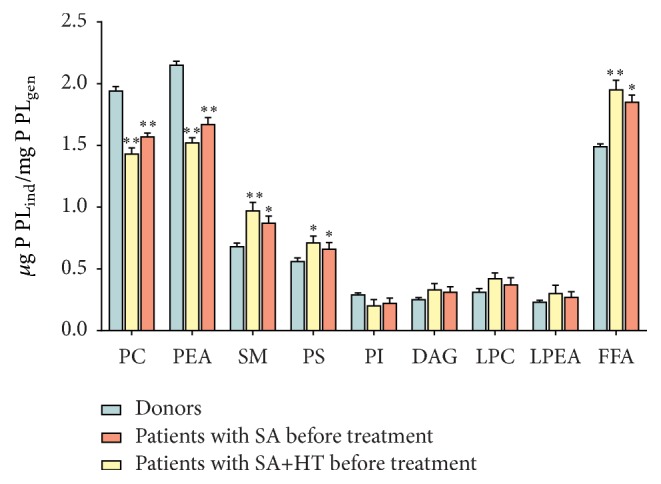
Change in the amount of phospholipids and lysophospholipids in red blood cells membranes of patients with stable angina (SA) and stable angina with hypertension (HT): *µ*g P  PL_ind_/mg P  PL_gen_. ^*∗∗*^ *p* < 0.01; ^*∗*^ *p* < 0.05.

The lipid composition of erythrocytic membranes in patients with stable angina has substantial differences: the content of PS, DAG, and SM increases by 18.7; 22.3; and 27.9%, and the level of PC, PEA, and PI reduces by 19.1; 22.5; and 24.3% (*p* < 0.05), respectively, compared to control values (Figure 1).

Concurrent with this the content of lysophosphatidylcholine (LPC), lysophosphatidylethanolamine (LPEA), and free fatty acids (FFA) increases by 20.8; 18.3; and 24.2% (*p* < 0.05), respectively, compared to control values (Figure 1).

Patients with stable angina on top of the primary arterial hypertension have marked changes in phospholipid composition of erythrocytic membranes. These differences appear as the increase in concentration of PS, SM, and DAG by 26.7; 42.6; and 32.3% and the decrease in concentration of PC, PEA, and PI by 26.1; 29.5; and 30.3% (*p* < 0.05), respectively, compared to control values (Figure 1). Also notably, the erythrocytes of patients with stable angina on top of hypertension have the increase in LPC, LPEA, and FFA by 25.8; 28.3; and 31.2% (*p* < 0.05), respectively, compared to control values (Figure 1).

**Figure 2:**
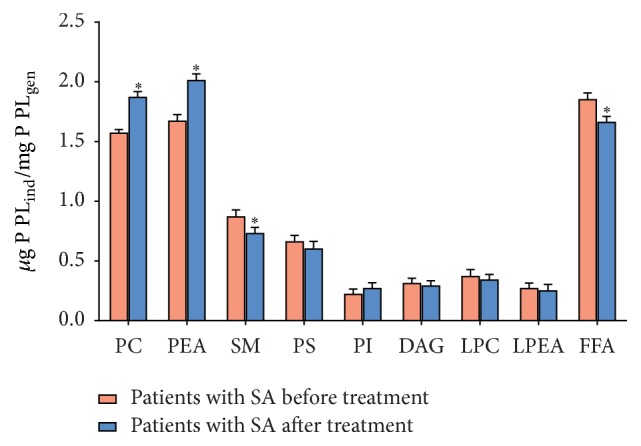
Change in the amount of phospholipids and lysophospholipids (*µ*g P  PL_ind_/mg P  PL_gen_) and free fatty acids (*µ*g P  PL_ind_/mg P  PL_gen_) in erythrocytic membranes of patients with stable angina (SA) before and after treatment. ^*∗*^ *p* < 0.05.

Depletion of erythrocytes by phosphatidylcholine that forms the outer shell of the lipid matrix points to the processes of disintegration of membrane structures, which may cause their destruction. Moreover, because PC demonstrates the properties of an inhibitor in processes of lipid peroxidation, its decreased amount in erythrocytes may lessen antioxidant protection of cell membranes [38]. The decrease in the amount of PEA and PC is apparently due to their partial hydrolysis by phospholipase A2, with simultaneous increase in monoacylated lysoderivatives—LPC and LFAA. A further proof to the activation of phospholipase A2 is the findings on the accumulation of FFA under the same conditions (Figure 2).

The decrease in the amount of PI in IHD patients can most likely be attributed to the activation of phospholipase C that causes PI degradation with the formation of inositol triphosphate and diacylglycerol, further metabolised into phosphatidic acid [39]. This is proven by our data on the increase in the content of DAG (Figure 1).

It is known that one of the important indicators of lipid biolayer condition is microviscosity, the parameters of which depend on the fatty acid composition of phospholipids that form erythrocytic membranes [40].

In all examined fractions of phospholipids, we found the increase in saturation ratio, which points to the decrease in the amount of unsaturated and the increase in saturated fatty acids. Moreover, the most marked changes occur in erythrocytes of patients with stable angina on a background of hypertension (Table 1).

Most likely, the increase in phospholipids lysoforms, as well as the change in the quantitative content and fatty acid composition of phospholipid fractions, is explained by the intensification of lipid peroxidation and activation of phospholipase [41, 42].

Apart from that, the increase in the number of PS may also point to the intensification of processes of LPO, as PS is the dominant activator of protein kinase C, the initiation of which in some cases may alternate with the intensification of and the weakening of antioxidant defense (AOD) [43].

**Figure 3:**
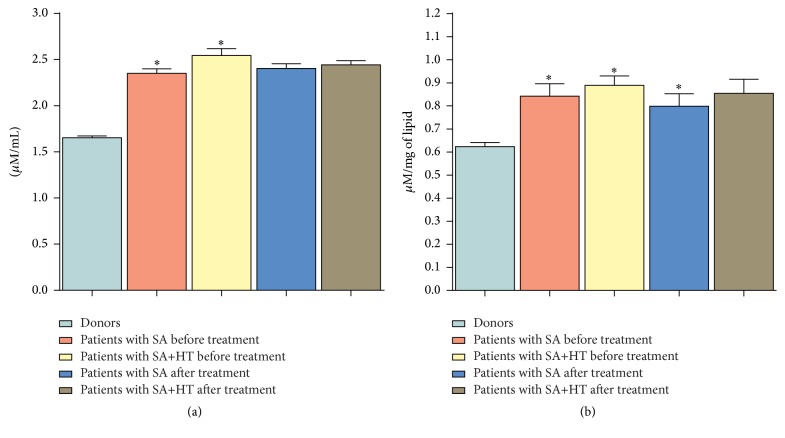
Change in the content of lipid peroxidation products in erythrocytic membranes of patients with SA and SA on the background of hypertension (HT) in the course of treatment. ^*∗*^ *p* < 0.05.

To confirm our hypothesis, we carried out a research where it was found that erythrocytes of patients with stable angina accumulate diene conjugates (DC) and malonic dialdehyde (MDA), the amount of which increases by 31.2 and 41.4% (*p* < 0.05), respectively, compared to control values. It should be noted that patients with stable angina on the background of hypertension demonstrate a more intensive activation of processes of lipid peroxidation (LPO): the level of DC and MDA in this variant of experiments increases by 38.9% and 52.6% (*p* < 0.05), respectively, compared with donors' erythrocytes (Figure 3).

The research into biomembranes' lipid composition of erythrocytes on the background of treatment revealed an increase in the amount of PC, PEA, and PI by 13.3; 15.8; and 16.7%, respectively, and the decrease in the amount of LPC and FFA by 12.2 and 12.5%, respectively, compared with blood of patients suffering from stable angina before treatment (Figures 1 and 2). Apart from that, the therapy carried out in this group facilitated the recovery of fatty acid composition in all studied fractions of phospholipids and the decrease in the content of DC and MDA (Table 1, Figure 3). It should be noted that in the group of patients with stable angina on the background of hypertension the treatment had no significant effect on the stabilisation of lipid composition of biomembranes of formed blood elements.

The change in PL, FA of phospholipids, the accumulation of FFA, DAG, and lysophospholipids observed in CVDs indicate that the lipid phase is the very medium that defines the structure and function of not only cell membranes, but also the cell in general [44]. The leading role here belongs to the lipolytic enzymes and oxidative processes in the lipid phase.

With modification of lipid composition and the change in the degree of unsaturation of fatty acid membranes the packing density of the lipid bilayer increases and microviscosity of their membranes improves. And therefore, it is possible to change the structure of erythrocyte.

The changes in morphology of the erythrocyte and, as a consequence, the distribution of hemoglobin in it significantly affect the efficiency of O_2_ transport within the body and is a sign of many pathologies, including cardiovascular disease [6].

**Figure 4:**
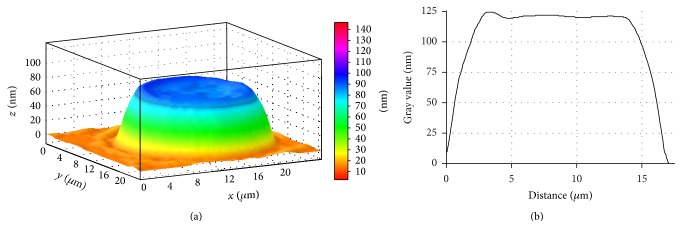
3D simulation (a) and a profile (b) of human erythrocyte in norm, LIM image.

**Figure 5:**
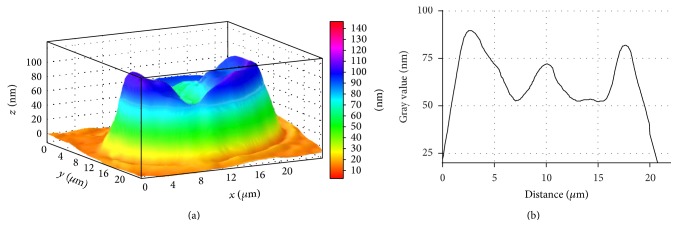
3D simulation (a) and phase profile (b) of SA patient's erythrocyte, LIM image.

Employing laser interference microscopy we examined the morphology of erythrocytes in patients with stable angina and patients with stable angina on the background of primary hypertension (Figures 4 and 5).

It is found that erythrocyte of a healthy donor has a normal discoidal shape, which is characterised by a uniform distribution of refraction index and, respectively, hemoglobin.

LIM imaging shows that the average the phase image area of erythrocyte equates to 110.92 ± 1.76 *µ*m^2^ and the erythrocyte volume makes 89.43 ± 2.37 *µ*m^3^.

Patients with stable angina have significant amounts of structurally altered erythrocytes (Figure 5). Phase images show that some erythrocytes, instead of a smooth doughnut shape, bear a “rough” shape with many irregularities (Figure 5). Most likely, the erythrocyte's rough shape in stable angina patients is caused by changes in cytoskeleton structure and hemoglobin redistribution in cytoplasm and submembranous areas.

These changes can be caused by pathological processes in erythrocytes developing in hypoxic conditions. The proof for the above is that the viscosity of the erythrocyte plasmatic membrane and property of hemoglobin changes in conditions of cardiovascular diseases [5]. It is the redistribution of lipids and, consequently, their charge on the surface which could affect the morphology of the erythrocyte. Besides, this image can also illustrate the cell's transition from discocyte to echinocyte.

**Figure 6:**
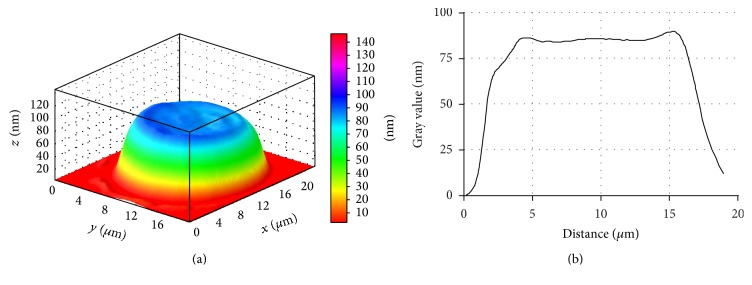
3D simulation (a) and phase profile (b) of SA patient's erythrocyte, LIM image. Stomatocyte.

It is found that the phase image area of erythrocyte decreases by 21.3% in SA patients. At the same time the erythrocyte volume increases by 42.2%, respectively, in relation to control value (Table 2, Figure 6).

**Figure 7:**
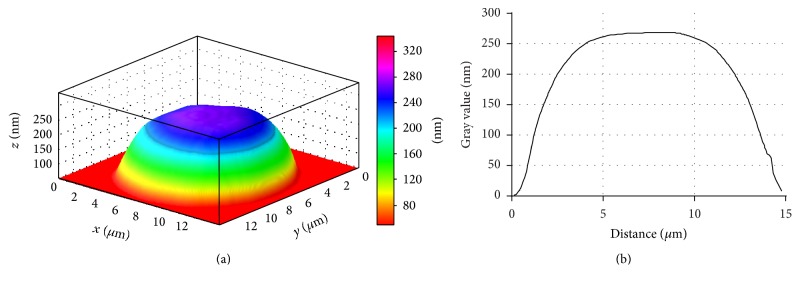
3D simulation (a) and phase profile (b) of SA patient's erythrocyte, LIM image. Spherocyte formation.

**Figure 8:**
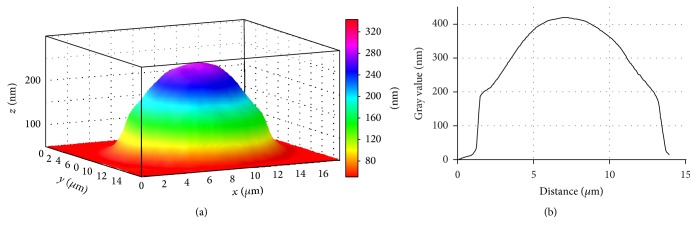
3D simulation (a) and phase profile (b) of SA+HP patients' erythrocyte, LIM image.

The erythrocytes can change their normal discoidal form and be transformed into echinocytes along with an increase in the area of their outer monolayer. Further transformation is possible into stomatocytes in conjunction with an increase in the area of the inner monolayer and into spherocytes. These changes can be partly caused by pathological processes in erythrocytes, including the condition when hypoxia develops, accompanying various manifestations of ischemic heart disease. It is now clear that structural discrepancies may be attributed to changes in the lipid composition of erythrocytic membranes (Figures 6, 7, and 8).

In addition to the findings above, in SA patients and SA+HT patients' blood, the quantity of irreversible erythrocyte shapes, spherocytes for instance, increased (Figures 7 and 8). These changes were significantly marked in cardiac angina without arterial hypertension.

The reversible transformation of a discocyte into an echinocyte begins with impaired biconcave erythrocyte structure contour with subsequent appearing of rough knots on the disk circumference and then on the whole cell surface after which the erythrocyte generally takes a spherical shape. Spherocyte formation represents the example of irreversible erythrocyte deformation (Figure 8).

Changes in the phase profile in different types of ischemic heart disease demonstrate redistribution and condensation of intracellular contents, firstly hemoglobin.

Standard treatment administered for these diseases does not bring any noticeable positive effect on normalisation of morphometric erythrocyte characteristics.

As changes of morphometric indicators will undoubtedly cause hemoglobin redistribution, it cannot fail to be reflected on its oxygen transforming functions determined in turn by conformation of hemoglobin hemoporphyrin.

Raman spectroscopy on the basis of hemoglobin hemoporphyrin spectra allows us to determine the oxidation degree of the included ferrum atom, its spin condition, and existence of ligands, reflecting the changes in globin structure leading to deformation of hemoporphyrin and influencing HB oxygen-binding properties.

The research into donors' erythrocytes and ischemic heart disease patients by means of RS showed changes in conformation of hemoglobin hematoporphyrin in conditions of hypoxia (Table 3).

Among other things found is the decrease in relative quantity of oxyhemoglobin (o-Hb) and relative ability of hemoglobin to isolate ligands for ischemic heart disease patients in comparison with healthy donors' indicators (by 28.9% and 46.5% for CA patients; by 40.7% and 37.9% for SA+HT patients, resp.).

Among other things found is the decrease in relative quantity of oxyhemoglobin (o-Hb) and relative ability of hemoglobin to isolate ligands for ischemic heart disease patients in comparison with healthy donors' indicators (by 28.9% and 46.5% for SA patients; by 40.7% and 37.9% for SA+HT patients, resp.).

Also we found the increase in relative ability of hemoglobin to bind ligands (including oxygen) and affinities of hemoglobin to ligands (first of all to oxygen) among SA patients (by 33.8% and 45.3% in comparison with donors' indicators) and SA with primary arterial hypertension patients (by 56.9% and 38.2% in comparison with donors' indicators).

Increased affinity of hemoglobin to oxygen and ability of hemoglobin to bind oxygen further intensifies the hypoxic processes already present when ischemic heart disease develops. Deterioration in ability of hemoglobin to release ligands including oxygen leads to the same thing.

It is possible to assume that the increased affinity of hemoglobin to oxygen and the arising hypoxia can be an important decisive pathogenetic sign of ischemic heart disease and arterial hypertension, as well as the cause of its exacerbation when hypertensive crises follow. The increased affinity of hemoglobin to oxygen reduces arteriovenous differences with reference to oxygen and, therefore, aggravates not only cerebral hypoxia, but also other organs and systems. Arising “vicious circles,” in their turn, can further worsen oxygen-transport function of blood [8]. Based on treatment results the erythrocytes of patients with ischemic heart disease (IHD) show better oxygen-binding capacity of hemoglobin, but in healthy people this capacity was in most cases much higher.

Therefore, to raise the efficiency of pathogenetic comprehensive treatment of patients with stable stenocardia and arterial hypertension, it is necessary to include medication relieving disturbances in the oxygen-transport function of blood.

## 4. Discussion

The results of our own research and review of literature on the topic prove that the change in physicochemical properties of the lipid bilayer of erythrocytic membranes largely determines the development of ischemic heart disease (IHD).

It is possible to identify a number of factors that influence the severity of the pathological processes in IHD: the accumulation of phospholipids' lysoforms (LPC and LPEA) and free fatty acids in the development of IHD may contribute to the “decondensation” of separate sections of the erythrocytic membrane lipid bilayer [45].

Significant increase in the number of FFA also affects the packing of bilayer integrity, influencing the increase in passive ion transport. All these processes impair gradients of ions concentration and, as a consequence, disturb erythrocytes' ionic balance [46].

Changes found in the content of PI and the simultaneous accumulation of DAG clearly point to the activation of the PI-cycle and, therefore, to the intensification of processes leading to the formation of secondary messengers which activate protein kinase C. This subsequently triggers phosphorylation and the activation of a large number of intracellular proteins.

This enhances Ca^2+^ permeability of erythrocytic membranes, which in its turn may trigger activation of Ca^2+^-dependent PLA2 [47].

The consequence of this is the strengthening of the destructive processes in the membrane of red blood cells in ischemic heart disease.

Changes of membrane lipid composition, in particular, the increase of PS, DAG, and SM and reduction of PC, PEA, and PI and the increase of lysoforms, trigger the disruption of protein-lipid interactions in the membrane and change the surface charge on the membrane.

In addition the change in the surface charge of the erythrocytic membrane can be attributed to experimentally found redistribution of charged fractions of phospholipids in patients with stable angina in favour of those neutral ones, located at the outer side of the plasma membrane (SM, LPC) and negatively charged PS, which is part of the inner surface of the membrane.

All these factors can also influence the transport of ions and change the oxygen-binding properties of the erythrocyte hemoglobin through the change in hematoporphyrin conformation [6, 25, 48–50].

Intensification of LPO processes, found in IHD patients' erythrocytes, firstly causes the decrease in the content of unsaturated fatty acids of phospholipids which is more marked in stable angina on the background of arterial hypertension. This triggers the increase in membranes' microviscosity and enhances their clusterisation. As a result the passive transport of ions including calcium ions increases due to the formation of nonspecific pores [51].

In the end, the intensification of LPO processes along with enzymatic degradation of PL contributes to membranes' damage and changes their physicochemical properties. As a result the disintegration of membrane structures culminates in their destruction and eventual cell death may occur [52, 53].

Numerous processes in the lipid bilayer membrane lead to changes in morphometric characteristics of erythrocytes among patients with IHD, namely, the appearance of echinocytes, stomatocytes, and spherocytes with redistribution and condensation of hemoglobin within the cell. The changes in morphology of the erythrocyte and, as a consequence, distribution of hemoglobin within it, significantly affect the O_2_ transport efficiency in the body and are signs of many pathologies, including cardiovascular diseases [6].

The revealed hemoglobin hematoporphyrin conformation disturbance in IHD patients' erythrocytes leads to the impaired oxygen-binding capacity of hemoglobin exemplified by the decrease in relative amount of oxyhemoglobin, relative ability of hemoglobin to drop ligands, the enhanced ability of hemoglobin to bind ligands, and the affinity of hemoglobin to ligands. This contributes to further intensification of the processes of hypoxia already present when IHD develops.

Therefore, the above processes occurring in the lipid bilayer of erythrocytic membranes affect the entire cytoarchitectonics of a cell, conformation of hemoglobin, and, consequently, the core function of erythrocytes—transport of oxygen. This mechanism is probably one of the most important phases in the development of ischemic heart disease.

The revealed changes in erythrocytes' properties may also worsen the tissue hypoxia and should be studied in further experiments.

## 5. Conclusion

The research findings showed that erythrocytic membranes, cytoarchitectonics of formed elements, lipids forming lipid membranes bilayer, and also hemoglobin represent a uniform highly dynamic system responding to the changes happening in the human body.

Based on findings and scientific literature it is possible to state that the prescribed therapy did not lead to normalisation of phospholipid and fatty acid structure of erythrocytic membranes. It requires a further search for natural compounds that can influence phospholipase activity and normalise processes of lipid peroxidation and, finally, the structure of hemoglobin. Traditional treatment does not affect one of the major phases of tissue oxygen supply or separate sites exposed to hypoxia. In order to advance the management of cardiovascular diseases it is necessary to take into account the possibility of administering medication improving the functional characteristics of both hemoglobin and erythrocytes.

## Figures and Tables

**Table 1:** The change in saturation ratio (*R*_sat_) of individual phospholipid fractions in erythrocytic membranes of patients with stable angina (SA) and SA with hypertension (HT) on the background of treatment (means ± SD).

Variants of experiments	*R*_sat_ PC, *µ*g P_pch_/mg P_fl_	*R*_sat_ PEA, *µ*g P_pea_/mg P_fl_	*R*_sat_ SM *µ*g P_sm_/mg P_fl_	*R*_sat_ PS *µ*g P_ps_/mg P_fl_	*R*_sat_ PI *µ*g P_pi_/mg P_fl_	*R*_sat_ DAG *µ*g P_dag_/mg P_fl_	*R*_sat_ FFA *µ*g P_ffa_/mg P_fl_
Donors	1.05 ± 0.021	1.3 ± 0.038	1.7 ± 0.021	1.2 ± 0.023	1.2 ± 0.021	1.95 ± 0.022	1.1 ± 0.02
Patients with SA before treatment	1.65 ± 0.06^*∗*^	2.86 ± 0.069^*∗*^	3.23 ± 0.055^*∗*^	1.8 ± 0.051	2.34 ± 0.056^*∗*^	2.71 ± 0.69^*∗*^	2.26 ± 0.094^*∗*^
Patients with SA+HT before treatment	1.77 ± 0.067^*∗*^	3.92 ± 0.066^*∗*^	3.84 ± 0.05^*∗*^	2.24 ± 0.044^*∗*^	2.87 ± 0.058^*∗*^	2.89 ± 0.061^*∗*^	3.21 ± 0.062^*∗*^
Patients with SA after treatment	1.29 ± 0.063^*∗∗*^	2.09 ± 0.082	2.58 ± 0.075^*∗∗*^	1.49 ± 0.06	1.62 ± 0.066	2.45 ± 0.089	1.58 ± 0.076^*∗∗*^
Patients with SA+HT after treatment	1.45 ± 0.047^*∗∗*^	3.02 ± 0.079^*∗∗*^	3.26 ± 0.026	1.93 ± 0.078	1.95 ± 0.078^*∗∗*^	2.64 ± 0.079	2.41 ± 0.043^*∗∗*^

^*∗*^Reliability in relation to donors' indicators.

^*∗∗*^Reliability in relation to pretreatment indicators at *p* < 0.05.

**Table 2:** Distribution of erythrocytes sizes in the course of SA patients' treatment and healthy persons (means ± SD).

Patients groups	Indicators		
	Phase image area of erythrocyte, *S*, *µ*m^2^	Erythrocyte volume, *V*, *µ*m^3^	The mean value of optical path difference (OPD), Φ_mean_, *n*_*m*_
Donors	110.92 ± 0.54	89.43 ± 3.63	60.92 ± 2.36
SA patients before treatment	87.35 ± 3.01^*∗*^	126.29 ± 3.15^*∗*^	108.44 ± 2.28^*∗*^
SA+HT patients before treatment	92.32 ± 4.77	109.21 ± 3.61	88.72 ± 4.30
SA patients after treatment	99.44 ± 2.45	118.47 ± 2.68^*∗∗*^	89.35 ± 1.92
SA+HT patients after treatment	101.30 ± 1.95	108.88 ± 2.04	80.61 ± 1.51

^*∗*^Reliability in relation to donors' indicators.

^*∗∗*^Reliability in relation to pretreatment indicators at *p* < 0.05.

**Table 3:** Changes of oxygen binding ability of hemoglobin in the course of treatment of IHD patients and healthy persons (means ± SD).

Patients groups	Variants			
	Relative quantity of o-Hb in blood, rel. Un. *I*_1375_/(*I*_1355_ + *I*_1375_)	Relative ability of Hb to bind ligands (including O_2_), rel. Un. *I*_1355_/*I*_1550_	Relative ability of Hb to isolate ligands, rel. Un. *I*_1375_/*I*_1580_	Affinity of Hb to ligands, first of all to O_2_, rel. Un. (*I*_1355_/*I*_1550_)/(*I*_1375_/*I*_1580_)
Donors	0.76 ± 0.02	0.65 ± 0.03	0.58 ± 0.02	1.28 ± 0.02

Patients before treatment				
SA patients before treatment	0.54 ± 0.03^*∗*^	0.87 ± 0.03^*∗*^	0.31 ± 0.02^*∗*^	1.86 ± 0.04^*∗*^
SA+HT patients before treatment	0.45 ± 0.03^*∗*^	1.02 ± 0.03^*∗*^	0.36 ± 0.02^*∗*^	1.77 ± 0.04^*∗*^

Patients after treatment				
SA patients after treatment	0.63 ± 0.03^*∗∗*^	0.73 ± 0.03	0.45 ± 0.03^*∗∗*^	1.26 ± 0.02^*∗∗*^
SA+HT patients after treatment	0.49 ± 0.08^*∗*^	0.75 ± 0.07^*∗∗*^	0.39+0.04	1.47 ± 0.04^*∗*^

^*∗*^Reliability in relation to donors' indicators.

^*∗∗*^Reliability in relation to pretreatment indicators at *p* < 0.05.

## References

[1] European Stroke Organisation (ESO) Executive Committee (2008). Guidelines for management of ischaemic stroke and transient ischaemic attack 2008. *Cerebrovascular Diseases*.

[2] Upadhyay R. K. (2015). Emerging risk biomarkers in cardiovascular diseases and disorders. *Journal of Lipids*.

[3] Pierson D. J. (2000). Pathophysiology and clinical effects of chronic hypoxia. *Respiratory Care*.

[4] Maksimov G. V., Rodnenkov O. V., Churin A. A., Rubin A. B., Tkachuk V. A., Chazov E. I. (2001). Influence of interval hypoxemic training on hemoglobin ability to bind oxygen in the blood of ischemia heart disease patient. *Cardiology*.

[5] Rodnenkov O. V., Luneva O. G., Ulyanova N. A. (2005). Erythrocyte membrane fluidity and haemoglobin haemoporphyrin conformation: Features revealed in patients with heart failure. *Pathophysiology*.

[6] Yusipovich A. I., Braze N. A., Luneva O. G. (2013). Changes in the state of hemoglobin in patients with coronary heart disease and patients with circulatory failure. *Bulletin of Experimental Biology and Medicine*.

[7] Revin V. V., Gromova N. V., Revina E. S. (2015). Study of the structure, oxygen-transporting functions, and ionic composition of erythrocytes at vascular diseases. *BioMed Research International*.

[8] Novitskiy V. V., Ryazantseva N. V., Stepovaya Ye. A. (2006). Molecular disorders of erythrocyte membrane due to pathology of different genesis are typical reaction of a body: contours of the problem. *Bulletin of Siberian Medicine*.

[9] Jewell S. A., Petrov P. G., Winlove C. P. (2013). The effect of oxidative stress on the membrane dipole potential of human red blood cells. *Biochimica et Biophysica Acta—Biomembranes*.

[10] Mohandas N., Gallagher P. G. (2008). Red cell membrane: past, present, and future. *Blood*.

[11] Salzer U., Prohaska R. (2001). Stomatin, flotillin-1, and flotillin-2 are major integral proteins of erythrocyte lipid rafts. *Blood*.

[12] Murphy S. C., Samuel B. U., Harrison T. (2004). Erythrocyte detergent-resistant membrane proteins: their characterization and selective uptake during malarial infection. *Blood*.

[13] Devyatkin A. A., Revin V. V., Yudanov M. A., Kozlova O. V., Samuilov V. D. (2006). Effect of hydrogen peroxide on ejection of cell nucleus from pigeon erythrocytes and state of membrane lipids. *Bulletin of Experimental Biology and Medicine*.

[14] Niki E. (2008). Lipid peroxidation products as oxidative stress biomarkers. *BioFactors*.

[15] Yoshida Y., Saito Y., Hayakawa M. (2007). Levels of lipid peroxidation in human plasma and erythrocytes: comparison between fatty acids and cholesterol. *Lipids*.

[16] Vassiljeva E. M. (2005). Biochemical peculiarity of the blood red cells. The influence of the pathology. A review. *Biomeditsinskaya Khimiya*.

[17] Gorbunov N. V. (1993). Effect of structural modification of membrane proteins on lipid-protein interactions in the human erythrocyte membranes. *Bulletin of Experimental Biology and Medicine*.

[18] Palsdottir H., Hunte C. (2004). Lipids in membrane protein structures. *Biochimica et Biophysica Acta—Biomembranes*.

[19] Bligh E. G., Dyer W. J. (1959). A rapid method of total lipid extraction and purification. *Canadian Journal of Biochemistry and Physiology*.

[20] Reich E., Schibli A. (2004). A standardized approach to modern high performance thin-layer chromatography (HPTLC). *Journal of Planar Chromatography*.

[21] Handloser D., Widmer V., Reich E. (2008). Separation of phospholipids by HPTLC—an investigation of important parameters. *Journal of Liquid Chromatography and Related Technologies*.

[22] Evans W. S., Morro D. D., O'Braytman E. (1990). *The Biological Membrane. Methods. (Biological Membranes. A Practical Approach)*.

[23] Morrison W. R., Smith L. M. (1964). Preparation of fatty acid methyl esters and dimethylacetals from lipids. *Journal of Lipid Research*.

[24] Brazhe N. A., Brazhe A. R., Pavlov A. N. (2006). Unraveling cell processes: interference imaging interwoven with data analysis. *Journal of Biological Physics*.

[25] Yusipovich A. I., Bryzgalova N. Y., Parshina E. Y. (2008). Evaluation of erythrocyte shape and status by laser interference microscopy. *Bulletin of Experimental Biology and Medicine*.

[26] Minaev V. L., Yusipovich A. I. (2012). Medical and biological measurements: use of an automated interference microscope in biological research. *Measurement Techniques*.

[27] Yusipovich A. I., Parshina E. Y., Brysgalova N. Y. (2009). Laser interference microscopy in erythrocyte study. *Journal of Applied Physics*.

[28] Brazhe A. R., Brazhe N. A., Sosnovtseva O. V., Pavlov A. N., Mosekilde E., Maksimov G. V. (2009). Wavelet-based analysis of cell dynamics measured by interference microscopy. *Computer Research and Modeling*.

[29] Yusipovich A. I., Zagubizhenko M. V., Levin G. G. (2011). Laser interference microscopy of amphibian erythrocytes: impact of cell volume and refractive index. *Journal of Microscopy*.

[30] Mityanina V. A., Parshina E. Y., Yusipovich A. I., Maksimov G. V., Selischeva A. A. (2012). Oxygen-binding characteristics of erythrocyte in children with type I diabetes mellitus of different duration. *Bulletin of Experimental Biology and Medicine*.

[31] Schindelin J., Arganda-Carreras I., Frise E. (2012). Fiji: an open-source platform for biological-image analysis. *Nature Methods*.

[32] Brazhe N. A., Abdali S., Brazhe A. R. (2009). New insight into erythrocyte through in vivo surface-enhanced Raman spectroscopy. *Biophysical Journal*.

[33] Brazhe N. A., Baizhumanov A. A., Parshina E. Y. (2014). Studies of the blood antioxidant system and oxygen-transporting properties of human erythrocytes during 105-day isolation. *Human Physiology*.

[34] Maksimov G. V., Brazhe N. A., Yusipovich A. I. (2011). Use of nanoparticles for studying the conformations of submembrane hemoglobin. *Biophysics*.

[35] Vlasov A. P., Trofimov V. A., Tarasova T. V. (2012). Structural-functional state of hemoglobin in gestosis. *Modern Problems of Science and Education*.

[36] Geary R. C. (1947). Testing for normality. *Biometrika*.

[37] Tukey J. W. (1949). Comparing individual means in the analysis of variance. *Biometrics*.

[38] Leskova G. F., Michunskaya A. B., Kobozeva L. P., Klimenko E. D. (2003). Influence of perftoran on structural and metabolic disturbances in the liver during experimental atherosclerosis. *Bulletin of Experimental Biology and Medicine*.

[39] Liscovitch M., Czarny M., Fiucci G., Tang X. (2000). Phospholipase D: molecular and cell biology of a novel gene family. *Biochemical Journal*.

[40] Prades J., Funari S. S., Escribá P. V., Barceló F. (2003). Effects of unsaturated fatty acids and triacylglycerols on phosphatidylethanolamine membrane structure. *Journal of Lipid Research*.

[41] Vladimirov Y. A. (2002). The loss of barrier properties by inner and outer mitochondrial membranes, necrosis and apoptosis. *Biologicheskie Membrany*.

[42] Shvedova A. A., Tyurina Y. Y., Tyurina V. A., Kikuchi Y., Kagan V. E., Quinn P. J. (2001). Quantitative analysis of phospholipid peroxidation and antioxidant protection in live human epidermal keratinocytes. *Bioscience Reports*.

[43] Kreps E. M. Lipidy klenochnykh membran (Lipids of Cell Membranes).

[44] Edwards C. J., Fuller J. (1996). Oxidative stress in erythrocytes. *Comparative Haematology International*.

[45] Mills J. K., Needham D. (2005). Lysolipid incorporation in dipalmitoylphosphatidylcholine bilayer membranes enhances the ion permeability and drug release rates at the membrane phase transition. *Biochimica et Biophysica Acta—Biomembranes*.

[46] Ibarguren M., López D. J., Escribá P. V. (2014). The effect of natural and synthetic fatty acids on membrane structure, microdomain organization, cellular functions and human health. *Biochimica et Biophysica Acta (BBA)—Biomembranes*.

[47] Woon L. A., Holland J. W., Kable E. P. W., Roufogalis B. D. (1999). Ca^2+^ sensitivity of phospholipid scrambling in human red cell ghosts. *Cell Calcium*.

[48] Shaklai N., Yguerabide J., Ranney H. M. (1977). Interaction of hemoglobin with red blood cell membranes as shown by a fluorescent chromophore. *Biochemistry*.

[49] Walder J. A., Chatterjee R., Steck T. L. (1984). The interaction of hemoglobin with the cytoplasmic domain of band 3 of the human erythrocyte membrane. *Journal of Biological Chemistry*.

[50] Chen Q., Balazs T. C., Nagel R. L., Hirsch R. E. (2006). Human and mouse hemoglobin association with the transgenic mouse erythrocyte membrane. *FEBS Letters*.

[51] Antonov V. F., Puchkov M. N., Korepanova E. A., Nemchenko O. Y., Borodulin V. (2014). Soft perforation of cardiolipin-containing planar lipid bilayer membrane by cytochrome c and H_2_O_2_. *European Biophysics Journal*.

[52] Pandey K. B., Rizvi S. I. (2010). Markers of oxidative stress in erythrocytes and plasma during aging in humans. *Oxidative Medicine and Cellular Longevity*.

[53] Revin V. V., Filatova S. M., Syusin I. V. (2015). Study of correlation between state and composition of lipid phase and change in erythrocytes structure under induction of oxidative processes. *International Journal of Hematology*.

